# Crystal structure of (*E*)-2-(2-{5-[(2-acet­oxy­eth­yl)(meth­yl)amino]­thio­phen-2-yl}vin­yl)-3-methyl­benzo­thia­zolium iodide monohydrate

**DOI:** 10.1107/S1600536814020121

**Published:** 2014-09-13

**Authors:** Xian-Shun Sun, Ming-Ming Wang, Dan-Dan Li

**Affiliations:** a Department of Chemistry, Anhui University, Hefei 230039, People’s Republic of China; b Key Laboratory of Functional Inorganic Materials Chemistry, Hefei 230039, People’s Republic of China

**Keywords:** crystal structure, benzo­thia­zolium iodide salt, hydrogen bonding

## Abstract

In the cation of the title hydrated salt, C_19_H_21_N_2_O_2_S_2_^+^·I^−^·H_2_O, the benzo­thia­zolium ring system is approximately planar [maximum deviation = 0.0251 (15) Å], and it makes a small dihedral angle of 1.16 (18)° with the plane of the thio­phene ring. In the crystal, the cations, anions and crystalline water mol­ecules are linked by classical O—H⋯O, O—H⋯I and weak C—H⋯O hydrogn bonds, forming a three-dimensional supra­molecular network. π–π stacking is observed between parallel thia­zole rings of adjacent cations [centroid–centroid distance = 3.5945 (16) Å].

## Related literature   

Interest in organic compounds with non-linear optical (NLO) properties is driven by the prospective of their applications in optical information technologies. The most common design of molecules with large NLO-activity comprises strong electron donors and acceptors connected by a π-conjugated system, see: Hao *et al.* (2009 ▶); Zhou *et al.* (2011 ▶). For the crystal structures of related benzo­thia­zolium derivatives, see: Quist *et al.* (2009 ▶).
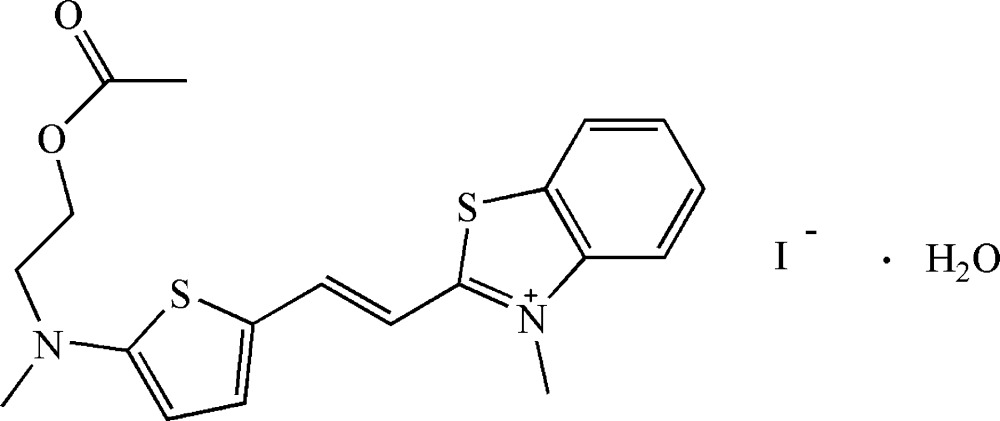


## Experimental   

### Crystal data  

C_19_H_21_N_2_O_2_S_2_^+^·I^−^·H_2_O
*M* *_r_* = 518.43
Triclinic, 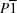

*a* = 9.6689 (9) Å
*b* = 11.1237 (11) Å
*c* = 11.1693 (11) Å
α = 94.420 (1)°
β = 110.067 (1)°
γ = 99.919 (1)°
*V* = 1099.40 (18) Å^3^
*Z* = 2
Mo *K*α radiation
μ = 1.67 mm^−1^
*T* = 293 K
0.20 × 0.20 × 0.18 mm

### Data collection  

Bruker SMART 1000 CCD area-detector diffractometer
8450 measured reflections
4211 independent reflections
3638 reflections with *I* > 2σ(*I*)
*R*_int_ = 0.016

### Refinement  

*R*[*F* ^2^ > 2σ(*F* ^2^)] = 0.028
*wR*(*F* ^2^) = 0.071
*S* = 0.97
4211 reflections
247 parameters
H-atom parameters constrained
Δρ_max_ = 0.67 e Å^−3^
Δρ_min_ = −0.30 e Å^−3^

### 

Data collection: *SMART* (Bruker, 2007 ▶); cell refinement: *SAINT* (Bruker, 2007 ▶); data reduction: *SAINT*; program(s) used to solve structure: *SHELXTL* (Sheldrick, 2008 ▶); program(s) used to refine structure: *SHELXTL*; molecular graphics: *SHELXTL*; software used to prepare material for publication: *SHELXTL*.

## Supplementary Material

Crystal structure: contains datablock(s) I, Global. DOI: 10.1107/S1600536814020121/xu5820sup1.cif

Structure factors: contains datablock(s) I. DOI: 10.1107/S1600536814020121/xu5820Isup2.hkl

**Fig. 1.**
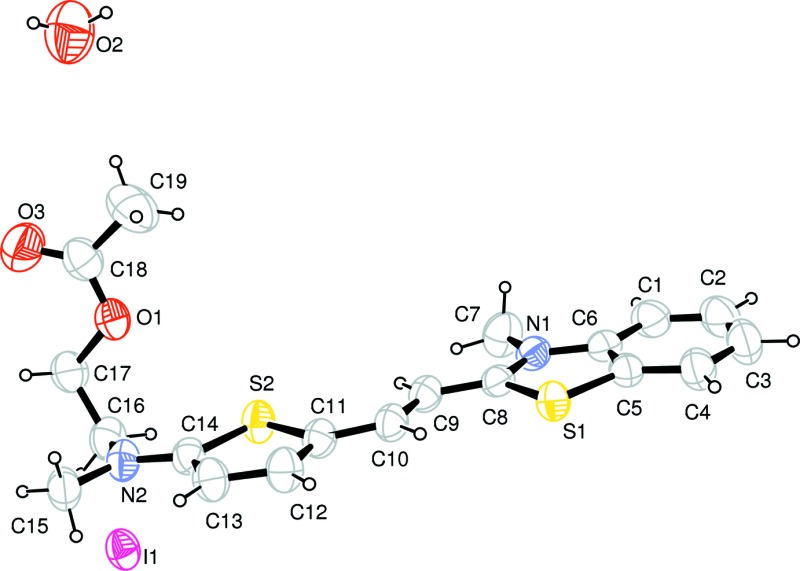
: The molecular structure of the title compound (I) showing 30% probability displacement ellipsoids.

. DOI: 10.1107/S1600536814020121/xu5820fig1.tif
The mol­ecular structure of the title compound (I) showing 30% probability displacement ellipsoids.

CCDC reference: 1023218

Additional supporting information:  crystallographic information; 3D view; checkCIF report

## Figures and Tables

**Table 1:** Hydrogen-bond geometry (Å, °)

*D*—H⋯*A*	*D*—H	H⋯*A*	*D*⋯*A*	*D*—H⋯*A*
O2—H2*A*⋯I1^i^	0.85	2.95	3.647 (3)	140
O2—H2*C*⋯O3^ii^	0.85	2.35	3.056 (5)	140
C7—H7*A*⋯O3^i^	0.96	2.46	3.364 (5)	157
C19—H19*A*⋯O2	0.96	2.60	3.554 (6)	173

Symmetry codes: (i) 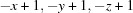; (ii) 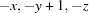.

## supplementary crystallographic information

### S1. Comment

Interest in organic compounds with non-linear optical (NLO) properties is driven by the prospective of their applications in optical information technologies. The most common design of molecules with large NLO-active comprises strong electron-donors and acceptors connected by a π-conjugated system (Hao *et al.*, 2009; Zhou *et al.*, 2011). Besides, the introduction about the highpolarizability of sulfur atoms in thiophene rings leads to a stabilization of the conjugated chain and to excellent charge transport properties. In the title compound (I) (Fig. 1), the benzothiazolium-CH═CH-thiophene part of the molecule is nearly coplanar (plane of CH═CH makes angles of 0.663 (8)° and 0.847 (1)° with the planes of the benzothiazolium and the thiophene rings), while in related benzothiazolium derivatives (Quist *et al.* 2009), the corresponding angles are 5.61 (18)° and 1.78 (19)°, respectively.

### S2. Experimental

A mixture of 2,3-dimethylbenzothiazolium iodide (1 mmol), 5-[(2-hydroxyethyl)methylamino]thiophene-2-carbaldehyde (1 mmol) and acetic anhydride (20 ml) was refluxed for 20 min, than poured into a warm solution of potassium iodide (4 mmol) in water (20 ml). The precipitated product was filtered, washed with water and recrystallized from a methanol/water solution. ^1^H NMR: (400 Hz, DMSO-d_6_), d(p.p.m.):8.14 (d, 1H), 8.11 (d, 1H), 7.89 (d, 1H), 7.83 (s, 1H), 7.66 (t, 1H), 7.53 (t, 1H), 6.73 (s, 1H), 6.54 (d, 1H), 4.33 (t, 2H), 4.00 (s, 3H), 3.85 (t, 2H), 3.24 (s, 3H), 1.94 (s, 3H).

### S3. Refinement

All hydrogen atoms were placed in geometrically idealized positions and constrained to ride on their parent atoms with O—H = 0.85, C—H = 0.93–0.97 Å, *U*_iso_(H) = 1.5*U*_eq_(C,O) for methyl H and water H atoms, and 1.2*U*_eq_(C) for the others.

### Crystal data

**Table:**

C_19_H_21_N_2_O_2_S_2_^+^·I^−^·H_2_O	*Z* = 2
*M**_r_* = 518.43	*F*(000) = 520
Triclinic, *P*1	*D*_x_ = 1.566 Mg m^−^^3^
Hall symbol: -P 1	Mo *K*α radiation, λ = 0.71073 Å
*a* = 9.6689 (9) Å	Cell parameters from 3223 reflections
*b* = 11.1237 (11) Å	θ = 2.6–26.8°
*c* = 11.1693 (11) Å	µ = 1.67 mm^−^^1^
α = 94.420 (1)°	*T* = 293 K
β = 110.067 (1)°	Block, red
γ = 99.919 (1)°	0.20 × 0.20 × 0.18 mm
*V* = 1099.40 (18) Å^3^	

### Data collection

**Table:**

Bruker SMART 1000 CCD area-detector diffractometer	3638 reflections with *I* > 2σ(*I*)
Radiation source: fine-focus sealed tube	*R*_int_ = 0.016
Graphite monochromator	θ_max_ = 26.0°, θ_min_ = 1.9°
phi and ω scans	*h* = −11→11
8450 measured reflections	*k* = −13→13
4211 independent reflections	*l* = −13→13

### Refinement

**Table:**

Refinement on *F*^2^	Primary atom site location: structure-invariant direct methods
Least-squares matrix: full	Secondary atom site location: difference Fourier map
*R*[*F*^2^ > 2σ(*F*^2^)] = 0.028	Hydrogen site location: inferred from neighbouring sites
*wR*(*F*^2^) = 0.071	H-atom parameters constrained
*S* = 0.97	*w* = 1/[σ^2^(*F*_o_^2^) + (0.036*P*)^2^ + 0.5766*P*] where *P* = (*F*_o_^2^ + 2*F*_c_^2^)/3
4211 reflections	(Δ/σ)_max_ < 0.001
247 parameters	Δρ_max_ = 0.67 e Å^−^^3^
0 restraints	Δρ_min_ = −0.30 e Å^−^^3^

### Special details

**Table:**

Geometry. All e.s.d.'s (except the e.s.d. in the dihedral angle between two l.s. planes) are estimated using the full covariance matrix. The cell e.s.d.'s are taken into account individually in the estimation of e.s.d.'s in distances, angles and torsion angles; correlations between e.s.d.'s in cell parameters are only used when they are defined by crystal symmetry. An approximate (isotropic) treatment of cell e.s.d.'s is used for estimating e.s.d.'s involving l.s. planes.
Refinement. Refinement of *F*^2^ against ALL reflections. The weighted *R*-factor *wR* and goodness of fit *S* are based on *F*^2^, conventional *R*-factors *R* are based on *F*, with *F* set to zero for negative *F*^2^. The threshold expression of *F*^2^ > σ(*F*^2^) is used only for calculating *R*-factors(gt) *etc*. and is not relevant to the choice of reflections for refinement. *R*-factors based on *F*^2^ are statistically about twice as large as those based on *F*, and *R*- factors based on ALL data will be even larger.

### Fractional atomic coordinates and isotropic or equivalent isotropic displacement parameters (Å^2^)

**Table:**

	*x*	*y*	*z*	*U*_iso_*/*U*_eq_	
C1	0.8392 (4)	1.1137 (3)	1.1880 (3)	0.0617 (8)	
H1	0.9151	1.1225	1.1540	0.074*	
C2	0.8380 (4)	1.2036 (3)	1.2804 (4)	0.0735 (10)	
H2	0.9138	1.2747	1.3076	0.088*	
C3	0.7274 (4)	1.1903 (3)	1.3330 (3)	0.0710 (9)	
H3	0.7318	1.2513	1.3968	0.085*	
C4	0.6105 (4)	1.0881 (3)	1.2926 (3)	0.0608 (8)	
H4	0.5353	1.0797	1.3274	0.073*	
C5	0.6088 (3)	0.9985 (2)	1.1986 (3)	0.0476 (6)	
C6	0.7227 (3)	1.0101 (3)	1.1481 (3)	0.0483 (6)	
C7	0.8124 (4)	0.8983 (3)	0.9953 (3)	0.0698 (9)	
H7A	0.7863	0.8181	0.9452	0.105*	
H7B	0.9112	0.9100	1.0600	0.105*	
H7C	0.8113	0.9604	0.9401	0.105*	
C8	0.5782 (3)	0.8216 (2)	1.0354 (2)	0.0432 (6)	
C9	0.5304 (3)	0.7106 (3)	0.9491 (3)	0.0481 (6)	
H9	0.5890	0.6925	0.9021	0.058*	
C10	0.4006 (3)	0.6289 (3)	0.9325 (3)	0.0485 (6)	
H10	0.3468	0.6512	0.9824	0.058*	
C11	0.3379 (3)	0.5172 (3)	0.8514 (3)	0.0498 (6)	
C12	0.2052 (3)	0.4370 (3)	0.8388 (3)	0.0565 (7)	
H12	0.1471	0.4533	0.8869	0.068*	
C13	0.1650 (3)	0.3322 (3)	0.7507 (3)	0.0553 (7)	
H13	0.0780	0.2720	0.7329	0.066*	
C14	0.2699 (3)	0.3259 (3)	0.6907 (3)	0.0481 (6)	
C15	0.1367 (4)	0.1291 (3)	0.5589 (3)	0.0627 (8)	
H15A	0.1256	0.0933	0.6314	0.094*	
H15B	0.1532	0.0685	0.5022	0.094*	
H15C	0.0469	0.1569	0.5134	0.094*	
C16	0.3834 (4)	0.2325 (3)	0.5509 (3)	0.0616 (8)	
H16A	0.4097	0.1522	0.5533	0.074*	
H16B	0.4725	0.2934	0.6045	0.074*	
C17	0.3371 (4)	0.2598 (3)	0.4147 (3)	0.0679 (9)	
H17A	0.4175	0.2563	0.3821	0.081*	
H17B	0.2482	0.1996	0.3597	0.081*	
C18	0.2295 (4)	0.4128 (3)	0.3042 (4)	0.0652 (9)	
C19	0.1969 (5)	0.5379 (4)	0.3189 (5)	0.1004 (14)	
H19A	0.1788	0.5703	0.2390	0.151*	
H19B	0.2815	0.5915	0.3852	0.151*	
H19C	0.1093	0.5326	0.3420	0.151*	
I1	0.77902 (2)	0.097609 (18)	0.70194 (2)	0.06164 (9)	
N1	0.7029 (3)	0.9082 (2)	1.0575 (2)	0.0474 (5)	
N2	0.2650 (3)	0.2336 (2)	0.6039 (2)	0.0543 (6)	
O1	0.3053 (3)	0.38152 (19)	0.4157 (2)	0.0676 (6)	
O2	0.0975 (4)	0.6590 (3)	0.0227 (3)	0.1165 (11)	
H2A	0.0901	0.7284	0.0548	0.175*	
H2C	0.0115	0.6207	−0.0300	0.175*	
O3	0.1931 (4)	0.3467 (3)	0.2039 (3)	0.1068 (10)	
S1	0.47604 (8)	0.86129 (6)	1.12820 (7)	0.04896 (17)	
S2	0.41617 (9)	0.45592 (7)	0.74660 (7)	0.05390 (18)	

### Atomic displacement parameters (Å^2^)

**Table:**

	*U*^11^	*U*^22^	*U*^33^	*U*^12^	*U*^13^	*U*^23^
C1	0.0601 (19)	0.0565 (18)	0.0615 (19)	0.0025 (15)	0.0177 (16)	0.0112 (15)
C2	0.076 (2)	0.0509 (19)	0.074 (2)	−0.0031 (17)	0.0110 (19)	0.0046 (16)
C3	0.081 (2)	0.0509 (18)	0.070 (2)	0.0110 (17)	0.0186 (19)	−0.0075 (16)
C4	0.068 (2)	0.0529 (17)	0.0599 (18)	0.0168 (15)	0.0211 (16)	0.0001 (14)
C5	0.0508 (16)	0.0426 (14)	0.0440 (15)	0.0127 (12)	0.0091 (12)	0.0068 (12)
C6	0.0536 (16)	0.0468 (15)	0.0409 (14)	0.0120 (13)	0.0110 (12)	0.0107 (12)
C7	0.066 (2)	0.082 (2)	0.066 (2)	0.0033 (18)	0.0367 (17)	0.0024 (17)
C8	0.0467 (15)	0.0472 (15)	0.0371 (13)	0.0148 (12)	0.0143 (11)	0.0091 (11)
C9	0.0509 (16)	0.0527 (16)	0.0423 (14)	0.0135 (13)	0.0187 (12)	0.0030 (12)
C10	0.0521 (16)	0.0505 (15)	0.0436 (15)	0.0154 (13)	0.0172 (12)	0.0015 (12)
C11	0.0511 (16)	0.0530 (16)	0.0466 (15)	0.0145 (13)	0.0192 (13)	0.0008 (12)
C12	0.0516 (17)	0.0603 (18)	0.0597 (18)	0.0108 (14)	0.0255 (14)	−0.0024 (14)
C13	0.0455 (16)	0.0543 (17)	0.0610 (18)	0.0055 (13)	0.0174 (14)	−0.0010 (14)
C14	0.0468 (15)	0.0492 (15)	0.0424 (14)	0.0137 (13)	0.0080 (12)	0.0025 (12)
C15	0.067 (2)	0.0493 (17)	0.0566 (18)	0.0091 (15)	0.0084 (15)	−0.0061 (14)
C16	0.0627 (19)	0.0632 (19)	0.0597 (18)	0.0244 (16)	0.0203 (15)	−0.0024 (15)
C17	0.097 (3)	0.0546 (18)	0.063 (2)	0.0304 (18)	0.0367 (19)	0.0014 (15)
C18	0.067 (2)	0.0576 (19)	0.076 (2)	0.0120 (16)	0.0336 (19)	0.0065 (18)
C19	0.114 (4)	0.069 (2)	0.126 (4)	0.039 (2)	0.042 (3)	0.019 (2)
I1	0.06545 (15)	0.05325 (13)	0.06963 (15)	0.00855 (9)	0.03333 (11)	−0.00352 (9)
N1	0.0494 (13)	0.0504 (13)	0.0425 (12)	0.0090 (11)	0.0174 (10)	0.0077 (10)
N2	0.0555 (14)	0.0523 (14)	0.0496 (13)	0.0115 (11)	0.0149 (11)	−0.0053 (11)
O1	0.0943 (17)	0.0500 (12)	0.0587 (13)	0.0227 (12)	0.0266 (12)	−0.0024 (10)
O2	0.121 (3)	0.121 (3)	0.106 (2)	0.028 (2)	0.044 (2)	−0.013 (2)
O3	0.150 (3)	0.093 (2)	0.0662 (17)	0.045 (2)	0.0181 (18)	−0.0002 (15)
S1	0.0494 (4)	0.0476 (4)	0.0501 (4)	0.0109 (3)	0.0197 (3)	−0.0004 (3)
S2	0.0526 (4)	0.0543 (4)	0.0534 (4)	0.0047 (3)	0.0237 (3)	−0.0065 (3)

### Geometric parameters (Å, º)

**Table:**

C1—C2	1.384 (5)	C12—C13	1.372 (4)
C1—C6	1.387 (4)	C12—H12	0.9300
C1—H1	0.9300	C13—C14	1.403 (4)
C2—C3	1.379 (5)	C13—H13	0.9300
C2—H2	0.9300	C14—N2	1.340 (3)
C3—C4	1.379 (5)	C14—S2	1.741 (3)
C3—H3	0.9300	C15—N2	1.459 (4)
C4—C5	1.385 (4)	C15—H15A	0.9600
C4—H4	0.9300	C15—H15B	0.9600
C5—C6	1.391 (4)	C15—H15C	0.9600
C5—S1	1.745 (3)	C16—N2	1.459 (4)
C6—N1	1.401 (3)	C16—C17	1.503 (5)
C7—N1	1.465 (4)	C16—H16A	0.9700
C7—H7A	0.9600	C16—H16B	0.9700
C7—H7B	0.9600	C17—O1	1.439 (4)
C7—H7C	0.9600	C17—H17A	0.9700
C8—N1	1.343 (3)	C17—H17B	0.9700
C8—C9	1.408 (4)	C18—O3	1.196 (4)
C8—S1	1.740 (3)	C18—O1	1.318 (4)
C9—C10	1.362 (4)	C18—C19	1.489 (5)
C9—H9	0.9300	C19—H19A	0.9600
C10—C11	1.387 (4)	C19—H19B	0.9600
C10—H10	0.9300	C19—H19C	0.9600
C11—C12	1.387 (4)	O2—H2A	0.8500
C11—S2	1.752 (3)	O2—H2C	0.8500
			
C2—C1—C6	117.4 (3)	C14—C13—H13	123.8
C2—C1—H1	121.3	N2—C14—C13	126.6 (3)
C6—C1—H1	121.3	N2—C14—S2	122.6 (2)
C3—C2—C1	121.7 (3)	C13—C14—S2	110.8 (2)
C3—C2—H2	119.2	N2—C15—H15A	109.5
C1—C2—H2	119.2	N2—C15—H15B	109.5
C2—C3—C4	121.1 (3)	H15A—C15—H15B	109.5
C2—C3—H3	119.4	N2—C15—H15C	109.5
C4—C3—H3	119.4	H15A—C15—H15C	109.5
C3—C4—C5	117.8 (3)	H15B—C15—H15C	109.5
C3—C4—H4	121.1	N2—C16—C17	112.6 (3)
C5—C4—H4	121.1	N2—C16—H16A	109.1
C4—C5—C6	121.1 (3)	C17—C16—H16A	109.1
C4—C5—S1	128.1 (3)	N2—C16—H16B	109.1
C6—C5—S1	110.7 (2)	C17—C16—H16B	109.1
C1—C6—C5	120.8 (3)	H16A—C16—H16B	107.8
C1—C6—N1	126.9 (3)	O1—C17—C16	107.5 (2)
C5—C6—N1	112.3 (2)	O1—C17—H17A	110.2
N1—C7—H7A	109.5	C16—C17—H17A	110.2
N1—C7—H7B	109.5	O1—C17—H17B	110.2
H7A—C7—H7B	109.5	C16—C17—H17B	110.2
N1—C7—H7C	109.5	H17A—C17—H17B	108.5
H7A—C7—H7C	109.5	O3—C18—O1	122.8 (3)
H7B—C7—H7C	109.5	O3—C18—C19	125.1 (4)
N1—C8—C9	126.3 (2)	O1—C18—C19	112.2 (3)
N1—C8—S1	111.84 (19)	C18—C19—H19A	109.5
C9—C8—S1	121.9 (2)	C18—C19—H19B	109.5
C10—C9—C8	121.8 (2)	H19A—C19—H19B	109.5
C10—C9—H9	119.1	C18—C19—H19C	109.5
C8—C9—H9	119.1	H19A—C19—H19C	109.5
C9—C10—C11	128.8 (3)	H19B—C19—H19C	109.5
C9—C10—H10	115.6	C8—N1—C6	114.3 (2)
C11—C10—H10	115.6	C8—N1—C7	123.9 (2)
C10—C11—C12	126.4 (3)	C6—N1—C7	121.8 (2)
C10—C11—S2	124.4 (2)	C14—N2—C15	119.5 (3)
C12—C11—S2	109.2 (2)	C14—N2—C16	122.8 (3)
C13—C12—C11	115.8 (3)	C15—N2—C16	117.7 (2)
C13—C12—H12	122.1	C18—O1—C17	117.4 (2)
C11—C12—H12	122.1	H2A—O2—H2C	109.5
C12—C13—C14	112.4 (3)	C8—S1—C5	90.83 (14)
C12—C13—H13	123.8	C14—S2—C11	91.88 (14)
			
C6—C1—C2—C3	1.2 (5)	C9—C8—N1—C7	−1.4 (4)
C1—C2—C3—C4	−2.0 (6)	S1—C8—N1—C7	178.5 (2)
C2—C3—C4—C5	0.8 (5)	C1—C6—N1—C8	−178.9 (3)
C3—C4—C5—C6	1.1 (4)	C5—C6—N1—C8	1.3 (3)
C3—C4—C5—S1	−179.4 (3)	C1—C6—N1—C7	2.1 (4)
C2—C1—C6—C5	0.6 (4)	C5—C6—N1—C7	−177.7 (3)
C2—C1—C6—N1	−179.2 (3)	C13—C14—N2—C15	3.2 (4)
C4—C5—C6—C1	−1.8 (4)	S2—C14—N2—C15	−178.3 (2)
S1—C5—C6—C1	178.6 (2)	C13—C14—N2—C16	−177.0 (3)
C4—C5—C6—N1	178.0 (3)	S2—C14—N2—C16	1.5 (4)
S1—C5—C6—N1	−1.6 (3)	C17—C16—N2—C14	−105.5 (3)
N1—C8—C9—C10	−179.3 (3)	C17—C16—N2—C15	74.3 (4)
S1—C8—C9—C10	0.7 (4)	O3—C18—O1—C17	−3.4 (5)
C8—C9—C10—C11	179.5 (3)	C19—C18—O1—C17	177.0 (3)
C9—C10—C11—C12	179.7 (3)	C16—C17—O1—C18	−164.4 (3)
C9—C10—C11—S2	−0.9 (5)	N1—C8—S1—C5	−0.4 (2)
C10—C11—C12—C13	179.2 (3)	C9—C8—S1—C5	179.6 (2)
S2—C11—C12—C13	−0.3 (4)	C4—C5—S1—C8	−178.4 (3)
C11—C12—C13—C14	0.8 (4)	C6—C5—S1—C8	1.1 (2)
C12—C13—C14—N2	177.7 (3)	N2—C14—S2—C11	−178.0 (2)
C12—C13—C14—S2	−1.0 (3)	C13—C14—S2—C11	0.7 (2)
N2—C16—C17—O1	61.2 (4)	C10—C11—S2—C14	−179.8 (3)
C9—C8—N1—C6	179.6 (3)	C12—C11—S2—C14	−0.3 (2)
S1—C8—N1—C6	−0.4 (3)		

### Hydrogen-bond geometry (Å, º)

**Table:**

*D*—H···*A*	*D*—H	H···*A*	*D*···*A*	*D*—H···*A*
O2—H2*A*···I1^i^	0.85	2.95	3.647 (3)	140
O2—H2*C*···O3^ii^	0.85	2.35	3.056 (5)	140
C7—H7*A*···O3^i^	0.96	2.46	3.364 (5)	157
C19—H19*A*···O2	0.96	2.60	3.554 (6)	173

Symmetry codes: (i) −*x*+1, −*y*+1, −*z*+1; (ii) −*x*, −*y*+1, −*z*.
