# Supplementary figures and images for: Crystal structure of (E)-2-(2-{5-[(2-acet­oxy­eth­yl)(meth­yl)amino]­thio­phen-2-yl}vin­yl)-3-methyl­benzo­thia­zolium iodide monohydrate
Source: Acta Crystallogr Sect E Struct Rep Online. 2014 Sep 13;70(Pt 10):o1104–5. doi: 10.1107/S1600536814020121 (PMC4257210; doi:10.1107/S1600536814020121)

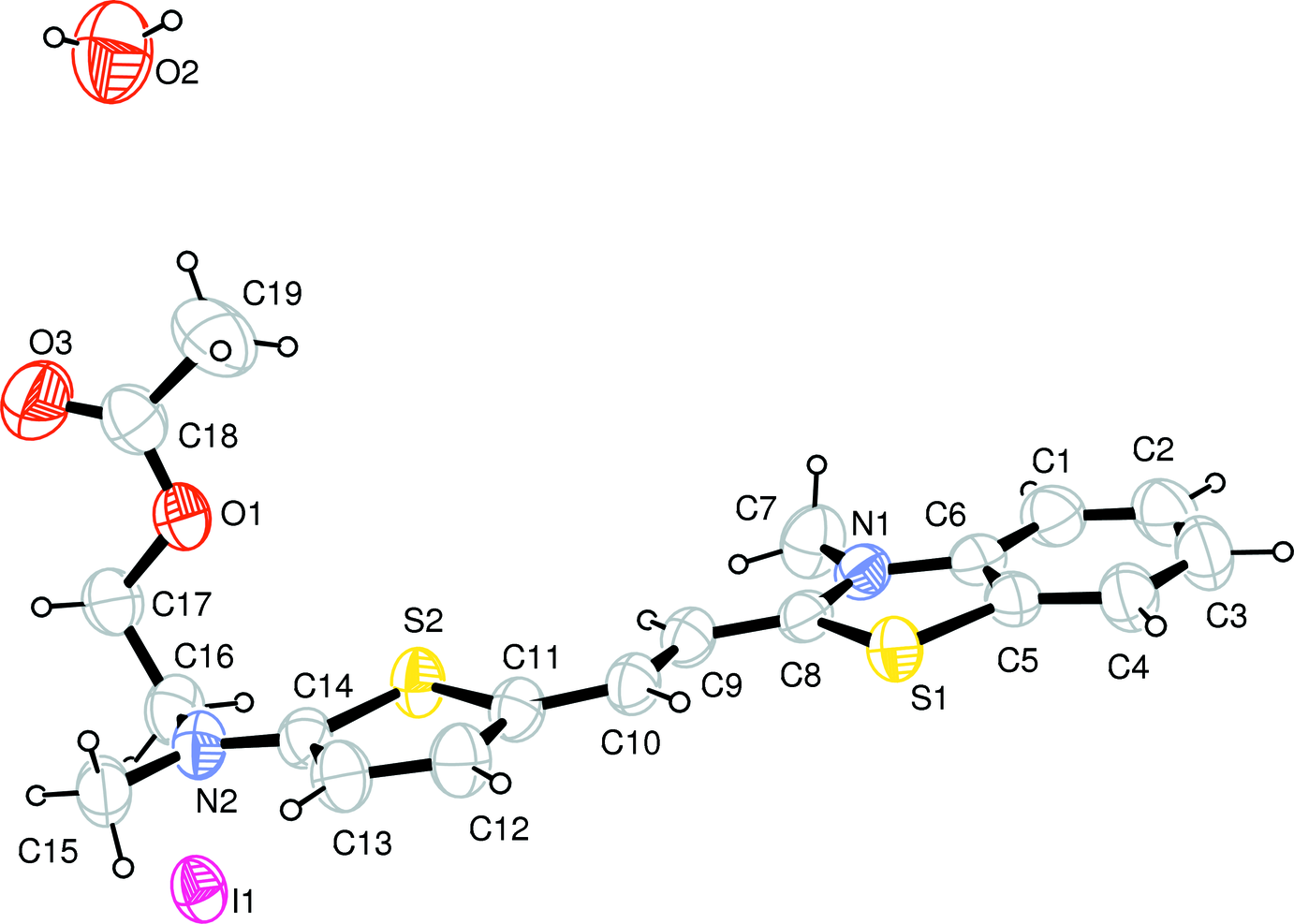

Supplement: Supplementary file 3 [file e-70-o1104-fig1.tif]
